# Genetic determinants of silver nanoparticle resistance and the impact of gamma irradiation on nanoparticle stability

**DOI:** 10.1186/s12866-024-03682-x

**Published:** 2025-01-13

**Authors:** Amira M. Mahfouz, Walaa A. Eraqi, Hala Nour El Din El Hifnawi, Alaa El Din Shawky, Reham Samir, Mohamed A. Ramadan

**Affiliations:** 1 Department of Drug Radiation Research, Division of Biotechnology, Laboratory of Drug Microbiology, National Center for Radiation Research and Technology (NCRRT), Egyptian Atomic Energy Authority (EAEA), Cairo, Egypt; 2 Department of Microbiology and Immunology, Faculty of Pharmacy, Cairo University, Kasr El-Aini Street, Cairo, 11562 Egypt

**Keywords:** Silver nanoparticles, Minimum inhibitory concentration, Whole genome sequencing and single nucleotide polymorphism

## Abstract

**Background:**

One of the main issues facing public health with microbial infections is antibiotic resistance. Nanoparticles (NPs) are among the best alternatives to overcome this issue. Silver nanoparticle (AgNPs) preparations are widely applied to treat multidrug-resistant pathogens. Therefore, there is an urgent need for greater knowledge regarding the effects of improper and excessive use of these medications. The current study describes the consequences of long-term exposure to sub-lethal concentrations of AgNPs on the bacterial sensitivity to NPs and the reflection of this change on the bacterial genome.

**Results:**

Chemical methods have been used to prepare AgNPs and gamma irradiation has been utilized to produce more stable AgNPs. Different techniques were used to characterize and identify the prepared AgNPs including UV-visible spectrophotometer, Fourier Transform Infrared (FT-IR), Dynamic light scattering (DLS), and zeta potential. Transmission electron microscope (TEM) and Scanning electron microscope (SEM) showed 50–100 nm spherical-shaped AgNPs. Eleven gram-negative and gram-positive bacterial isolates were collected from different wound infections. The minimum inhibitory concentrations (MICs) of AgNPs against the tested isolates were evaluated using the agar dilution method. This was followed by the induction of bacterial resistance to AgNPs using increasing concentrations of AgNPs. All isolates changed their susceptibility level to become resistant to high concentrations of AgNPs upon recultivation at increasing concentrations of AgNPs. Whole genome sequencing (WGS) was performed on selected susceptible isolates of gram-positive *Staphylococcus lentus* (St.L.1), gram-negative *Klebsiella pneumonia* (KP.1), and their resistant isolates St.L_R.Ag and KP_R.Ag to detect the genomic changes and mutations.

**Conclusions:**

For the detection of single-nucleotide polymorphisms (SNPs) and the identification of all variants (SNPs, insertions, and deletions) in our isolates, the Variation Analysis Service tool available in the Bacterial and Viral Bioinformatics Resource Center (BV-BRC) was used. Compared to the susceptible isolates, the AgNPs-resistant isolates St.L_R.Ag and KP_R.Ag had unique mutations in specific efflux pump systems, stress response, outer membrane proteins, and permeases. These findings might help to explain how single-nucleotide variants contribute to AgNPs resistance. Consequently, strict regulations and rules regarding the use and disposal of nano waste worldwide, strict knowledge of microbe-nanoparticle interaction, and the regulated disposal of NPs are required to prevent pathogens from developing nanoparticle resistance.

**Supplementary Information:**

The online version contains supplementary material available at 10.1186/s12866-024-03682-x.

## Introduction

Antimicrobial resistance (AMR) represents a top global threat to healthcare systems and national economies in the 21st century [1]. The fact that AMR was responsible for 1.27 million deaths worldwide in 2019 [2]. Several groups are at higher risk including, immunocompromised patients or those with underlying conditions such as diabetes, cancer, autoimmune disease, respiratory disorders, Human immune deficiency disease HIV, or aging [3–5]. The World Health Organization (WHO) adopted a strategy to raise awareness of AMR by launching surveillance systems [6] and implementing antimicrobial stewardship programs to cut down inappropriate use of antibiotics and improve access to appropriate treatment. The 2022 Global Antimicrobial Resistance and Use Surveillance System highlights alarming resistance rates among pathogenic bacteria including methicillin-resistant *Staphylococcus aureus*, cephalosporin-resistant *Escherichia coli*, and multidrug-resistant *Klebsiella pneumoniae* [7]. Multiple antibiotic discoveries have been reported since the use of the first antibiotic, penicillin. Regretfully, the emergence of antimicrobial resistance outpaces the reveal or development of novel antimicrobial treatments [8].

Nanomaterials have recently emerged as a weapon against bacteria resistant to many drugs. Metal and metal oxide nanoparticles (NPs) are one of the most studied nanomaterials against multidrug-resistant bacteria [9]. Metals such as silver, titanium, gold, aluminum, copper, and zinc can be used to create such NPs. Numerous evaluations have emphasized the significance of nanotechnology, which is undoubtedly incredibly versatile and has great potential in combating microbial diseases [10, 11]. The healthcare industry has been greatly impacted by the antimicrobial ability of AgNPs, which are used to create bactericidal coatings for medical equipment. Additionally, they can be found in cosmetics, packaging materials, and textiles [12].

The preparation of AgNPs was conducted through physical and chemical procedures [13]. The utilization of gamma radiation-induced techniques for NP synthesis offers distinct advantages over traditional chemical methods [14]. These advantages include the ability to control the shape and size of NPs, the use of non-toxic or low-toxic precursors along with environmentally friendly solvents, reduced reliance on chemical reagents, minimal generation of hazardous waste, and limited formation of reaction byproducts. This radiation-induced approach presents a more environmentally friendly pathway for NP preparation [15].

A significant proportion of antibiotic resistance mechanisms become redundant due to the direct binding of NPs to bacterial cell walls, inducing the bactericidal effect without the need to breach the cell membrane. Consequently, the effectiveness of NPs against bacteria is more likely to surpass that of antibiotics [16]. One of three models explains the antibacterial mechanism of action of NPs against bacterial isolates, oxidative stress induction [17], nonoxidative processes [18] and metal ion release [19]. Nevertheless, prolonged exposure to sub-lethal biocidal levels may induce efflux pump over-expression and the emergence of bacterial resistance [20]. Numerous harmful bacteria have already been found to be resistant to silver ions. Regretfully, current research has shown that the development of microbial resistance has also been facilitated by the chemical adaptability of NPs [21, 22].

Next generation sequencing (NGS) technology is becoming more and more common for WGS. With a unified, efficient workflow, NGS offers a fast, high throughput technology. One can utilize this technique to identify single-base pair mutations in bacteria belonging to the same species [23]. By offering a comprehensive database of genetic polymorphisms, especially single-nucleotide polymorphisms (SNPs), this method offers higher sequence resolution than conventional techniques. Additionally, WGS links pathogen biology, genome structure, genome evolution, and gene content to epidemiology. This connection sheds light on biological markers including virulence factors and antibiotic resistance [24].

Finding SNPs in bacterial genomes is important for tracking the evolution of resistant clinical isolates and establishing the relationship between them. Furthermore, genome sequencing simplifies the process of screening for specific changes or alterations within resistant genes when comparing them to susceptible or control strains of the same bacterial species [25, 26].

Unfortunately, recent research has shown that the chemical adaptability of NPs has also played a role in the rise of microbial resistance [21]. This study provides insights into the emerging microbial diseases that can induce resistance to NPs and pose a serious risk to human health. This study scrutinized single-nucleotide alterations within AgNPs-resistant and nonresistant gram-positive and gram-negative bacterial isolates. The nsSNPs identified in the AgNPs-resistant isolates have the potential to change the amino acid sequence, potentially impacting the function of the resulting protein expression. This alteration can provoke diverse resistance mechanisms.

## Materials and methods

### Preparation of AgNPs

Silver nanoparticles were produced by chemical reduction as previously described by Turkevich, Lee, and Meisel [27, 28]. A solution of 5 × 10^− 3^ M silver nitrate AgNO_₃_ (100 mL) was used as Ag⁺^₁^ ions precursor. It was added portion-wise to 300 mL of vigorously stirred ice-cold 2 × 10^− 3^ M NaBH_4_ (Sodium borohydride) as a mild reducing agent. A solution of 1% polyvinyl pyrrolidone (PVP) (50 mL) was added during the reduction as a stabilizing agent. The mixture was then boiled for 1 h to decompose any excess of NaBH_4_. The final volume was adjusted to 500 mL. As the Ag⁺ ions were reduced to Agº NPs, the solution’s color gradually changed to a yellowish-brown.

### Effect of gamma irradiation on AgNPs

Silver nanoparticles were irradiated with 5 KGy and 10 KGy gamma irradiation using a Cobalt 60 source (Gamma cell 4000-A-India) at room temperature. A non-irradiated sample was used as a control. The irradiation procedure was done at the National Center for Radiation Research and Technology (NCRRT).

### Characterization of AgNPs

After gamma irradiation, physicochemical characterization was performed using UV-visible spectrophotometer (JASCO V-560 UV/Vis, Japan) as a function of wavelength in the range of 200–900 nm, operating at a resolution of 1 nm. Additionally, Fourier Transform Infrared Spectroscopy (FT-IR) spectra were recorded using an infrared spectrometer (JASCO FT/IR-3600, Japan) in the range of 500–4000 cm^− 1^ using the KBr pellet technique. The FT-IR analysis examined the changes in AgNPs before and after exposure to gamma irradiation. The average particle size, size distribution, and zeta potential were evaluated by the PSS-NICOMP 380-ZLS particle sizing system (St. Barbara, California, USA) at NCRRT. The size and shape of the prepared NPs were documented by using the transmission electron microscope (TEM) model JEOL electron microscope (JEM-100 CX, Japan), at NCRRT. Drop-coating NPs onto carbon-coated TEM grids was used to prepare the grids for TEM. After allowing the film on the TEM grids to dry, the excess solution was wiped off with blotting paper. The surface morphologies and size of the NPs were examined by Scanning Electron Microscope (SEM) (ZEISS-EVO-MA10, Germany) attached with energy-dispersive X-ray spectra (EDX-BRUKER Nano GmbH, D-12489,410-M, Berlin, Germany) to detect the basic makeup of the prepared AgNPs.

### Bacterial isolation and identification

Eleven bacterial isolates were recovered from wound infections. Protein fingerprinting was performed using Matrix-Assisted Laser Desorption/Ionization Time-Of-Flight Mass Spectrometry (MALDI-TOF MS) with the Microflex MALDI-TOF MS system (Bruker Daltonics, Bremen, Germany) at 57,357 Hospital in Cairo, Egypt. The analysis and comparison were conducted using MALDI BioTyper 2.0 software and the MALDI BioTyper database, with species-level identification determined by a score of ≥ 2.0 according to the manufacturer’s criteria. Isolates were preserved in Muller Hinton Broth with 30% v/v glycerol and stored at -80ºC until needed [29, 30].

### MIC determination of AgNPs against the tested bacterial isolates

The minimum Inhibitory Concentration of AgNPs was determined by the Agar Dilution method according to the Clinical and Laboratory Standard Institute CLSI 2011 & 2018 [31, 32]. Bacterial isolates were grown overnight on Muller Hinton Agar (MHA) (HiMedia) plates at 35 °C before being used. Two-fold serial dilutions of AgNPs (0.1–100 µg/ml) were prepared and incorporated into the agar medium, with each plate containing a different concentration of the AgNPs. 0.1 µL were spot inoculated over the circles marked on agar plates from an initial bacterial density of each microbial isolate 1 × 10^8^ CFU/ml. After incubation for 24 h at 37˚C, the MIC was visually estimated on the surface of MHA plates. The MIC is the lowest concentration of antimicrobial agents that completely visually inhibits the 99% growth of the microorganisms. All the experiments were done in triplicates on three different days. All isolates were utilized as positive controls on MHA media without AgNPs.

### Induction and development of bacterial resistance to AgNPs

Induction of bacterial resistance was attempted by repeatedly culturing the isolates in growth media with sub-lethal doses of AgNPs, at concentrations sub-MIC where growth was detected. Each bacterial isolate underwent twenty successive cultivation steps in media containing sub-MIC of AgNPs. AgNPs stock solutions were prepared at concentrations ranging from 1.25 to 100 µg/ml, and long-term exposure involved gradually increasing the MIC of AgNPs. The MIC values after the 20th passage were visually estimated on the surface of MHA plates after 24 h of incubation and compared to the MIC values before the passages. The stability of AgNPs resistance after the 20th passage was assessed by subculturing the resistant isolates daily for more five passages. All isolates were used as negative controls in each passage, using MHA media without AgNPs.

### DNA extraction and whole genome sequencing

DNA of four bacterial isolates (gram-positive *Staphylococcus lentus* St.L.1, gram-negative *Klebsiella pneumonia* KP.1, and their AgNPs resistant isolates St.L_R.Ag and KP_R.Ag, respectively) were extracted using QIAamp DNA Mini Kit (Qiagen, Germany) according to the manufacturer’s protocol. The concentration of the extracted DNA was determined with a Qubit 3.0 Fluorometer and the HSDNA Qubit™ Assay Kit (Q32854, ThermoFisher Scientific Inc, USA). Bacterial DNA samples at a concentration of 20 ng/µl were done at Clinilab (Cairo, Egypt) for whole-genome sequencing.

### Sequencing, preprocessing, assembly, and genome annotation

The extracted DNAs were sequenced using the Ion torrent proton platform, Thermofischer Scientific Inc, USA. Single–end chemistry was used with an average library insert size 150–200 bp. The DNA libraries preparation from the extracted DNAs were performed using Ion Xpress™ plus fragment Library Kit, cat no. 4,471,269, Thermofischer Scientific Inc, USA as per the manufacturer’s instructions. Adapter sequences and low-quality reads were removed with a quality score filter of > 30. DNA libraries were quantified using Ion Library TaqMan™ Quantification kit, cat no. 4,468,802. Template preparation kit and sequencing were performed using Ion PI™ HI-Q™ Chef Kit (cat. No. A27198), Thermofischer Scientific Inc, USA.

High-quality sequence reads were de novo assembled using SPAdes assembler (v3.12.0), which is part of the Bacterial and Viral Bioinformatics Resource Center (BV-BRC/ PATRIC database), (https://www.bv-brc.org/) [33] with a minimum contig length of 300 bp.The assemblies were mapped against the control isolate to evaluate the core genome average identities and completeness. Assembled FASTA files were annotated by the BV-BRC (version 3.27.0) annotation service which uses the RASTtk (Rapid Annotation using Subsystem Technology) algorithm server for gene predictions and annotations [34, 35]. The prediction relied on the existing annotation resources such as Coding DNA Sequences (CDS) and proteins.

### Genome comparisons and analysis of single nucleotide polymorphisms

The Proteome Comparison Service available in BV-BRC was used to perform protein sequence-based genome comparison between the AgNPs resistant isolates and their corresponding reference genomes using bidirectional BLASTP. The proteome comparison results are displayed as an interactive circular genome view with color-coding for protein percent identity relative to the best hit on the reference genome.

For the detection of SNPs and the identification of all variants (SNPs, insertions, and deletions) in the tested isolates, we used the Variation Analysis Service tool available in BV-BRC. The sequencing reads of the AgNPs resistant isolates KP_R.Ag and St.L_R.Ag were mapped to their corresponding reference genome KP.1 and St.L.1 respectively, using LAST software (Frith et al. 2010). High-confidence SNP variants data sets were created by FreeBayes SNP caller (Marth et al. 1999) by applying a series of filters. The variants were identified and extracted using the following Parameters: the raw SNPs are filtered by SNP quality (QUAL > 10) and read depth (DP > 5) to keep only the high-quality SNPs.

### Data Availability

For the two *Klebsiella pneumonia* isolates (KP.1 & KP_R.Ag) and the two *Staphylococcus lentus* isolates (St.L.1 & St.L_R.Ag) used in this study, their genome assemblies have been deposited in the National Center for Biotechnology Information (NCBI) under the Bioproject accession number: PRJNA1108055. All raw sequences were deposited in the Sequence Read Archive database under the Bioproject accession number PRJNA1109764 (https://www.ncbi.nlm.nih.gov/sra/PRJNA1109764).

### Statistical methods

The mean MIC and results from antimicrobial susceptibility testing were imported into the Statistical Package for the Social Sciences (SPSS) software, version 20.0 (SPSS Inc., Chicago, IL, USA).

## Results

### Preparation, irradiation using gamma radiation, and characterization of AgNPs

**Fig. 1:**
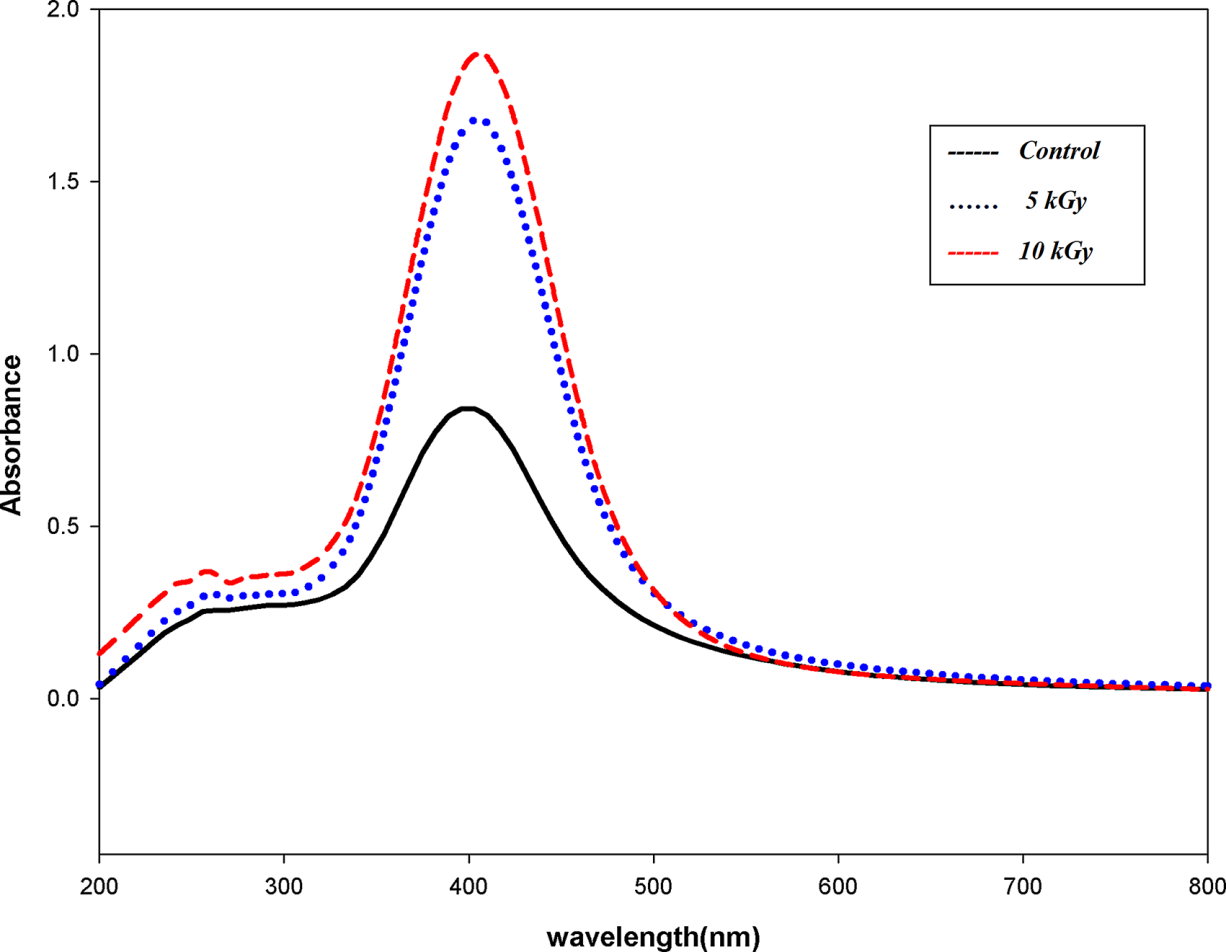
A curve showing UV-visible spectrophotometer analysis of the prepared silver nanoparticles (AgNPs) before and after 5 kGy and 10 kGy gamma irradiation. All samples show wavelength peaks at 400 nm. High absorbance peaks after gamma irradiation showing 1.7 and 1.9 at 5 and 10 kGy respectively indicating increase in NPs concentrations

The chemical synthesis of AgNPs resulted in a yellowish-brown solution. AgNPs were exposed to 5 kGy and 10 kGy gamma irradiation exposure levels. The structural assessment of AgNPs was done by analyzing the NPs before and following exposure to gamma irradiation using UV-visible spectrophotometry and FTIR analysis techniques. The UV-visible spectrophotometric analysis of unirradiated and irradiated AgNPs showed a wavelength peak value near 400 nm (Fig. 1**)**. After gamma irradiation, the UV analysis of AgNPs was characterized by a strong Plasmon band at 1.7 and 1.9 after 5 kGy and 10 kGy, respectively. The high Plasmon band after exposure to gamma irradiation indicated an increase in the concentration of the NPs. The chemical groups that are near AgNPs and contribute to their stability were examined by FTIR analysis. It also made it possible for us to ascertain whether the radiolytic reduction brought on by gamma irradiation had altered the functional groups in any way. The FTIR spectrum of AgNPs (Supplementary Figure S1a) showed inorganic bands 1500 –500 cm^− 1^ indicating an incomplete reduction of Ag^+^ ions in the solution. After gamma irradiation, no functional transformation was found and the inorganic band at 588 cm^− 1^ indicated a complete decrease of Ag^+^ ions to Agº and a complete stabilization of AgNPs (Supplementary Figures S1b & S1c).

**Fig. 2:**
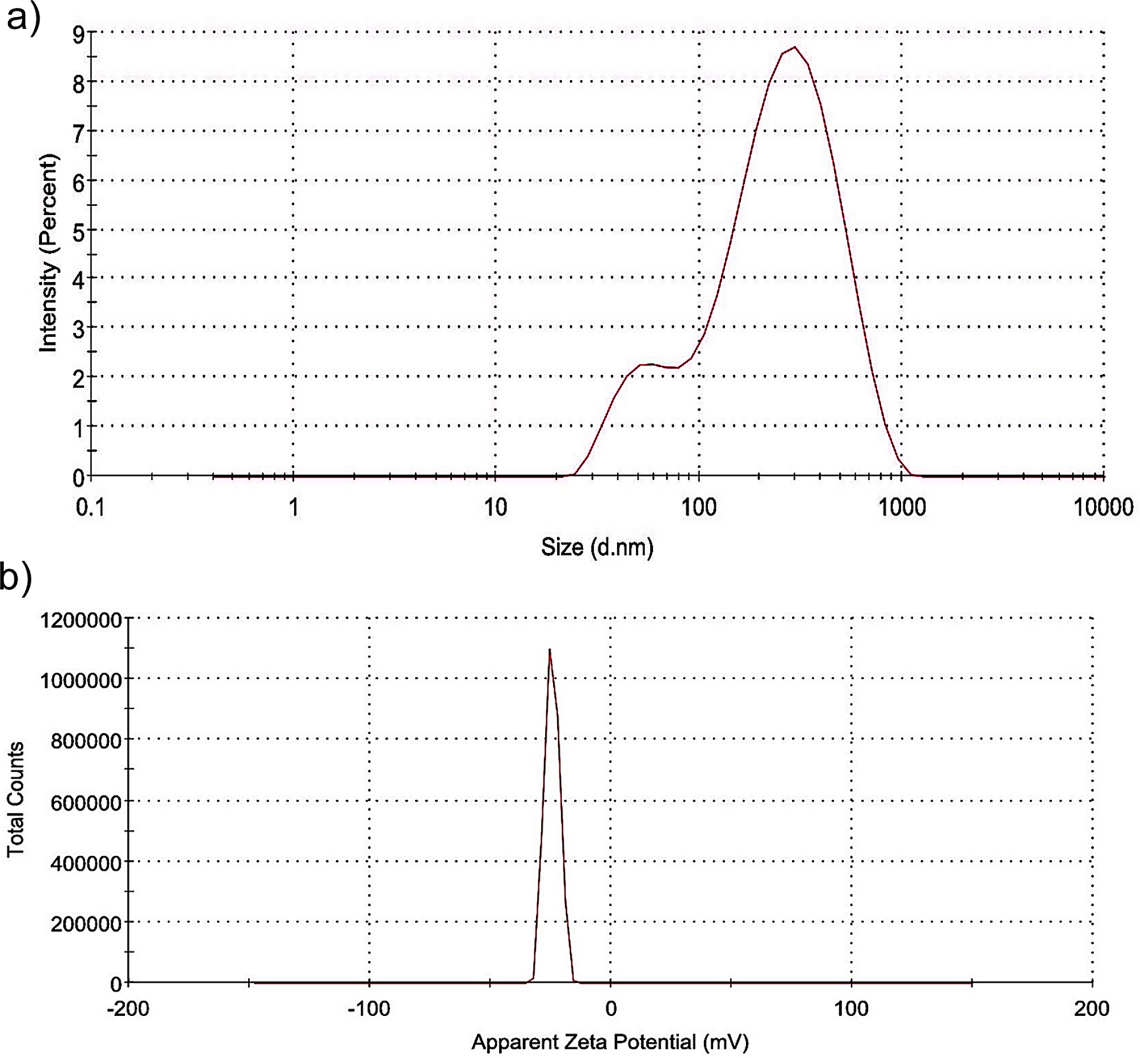
Dynamic light scattering (DLS) and Zeta potential of the prepared silver nanoparticles (AgNPs): **a**) Particle size Distribution by DLS of the prepared AgNPs showing average particle size 50–500 nm **b**) Zeta potential for the prepared AgNPs showing – 24.4 mV indicating high stability

**Fig. 3:**
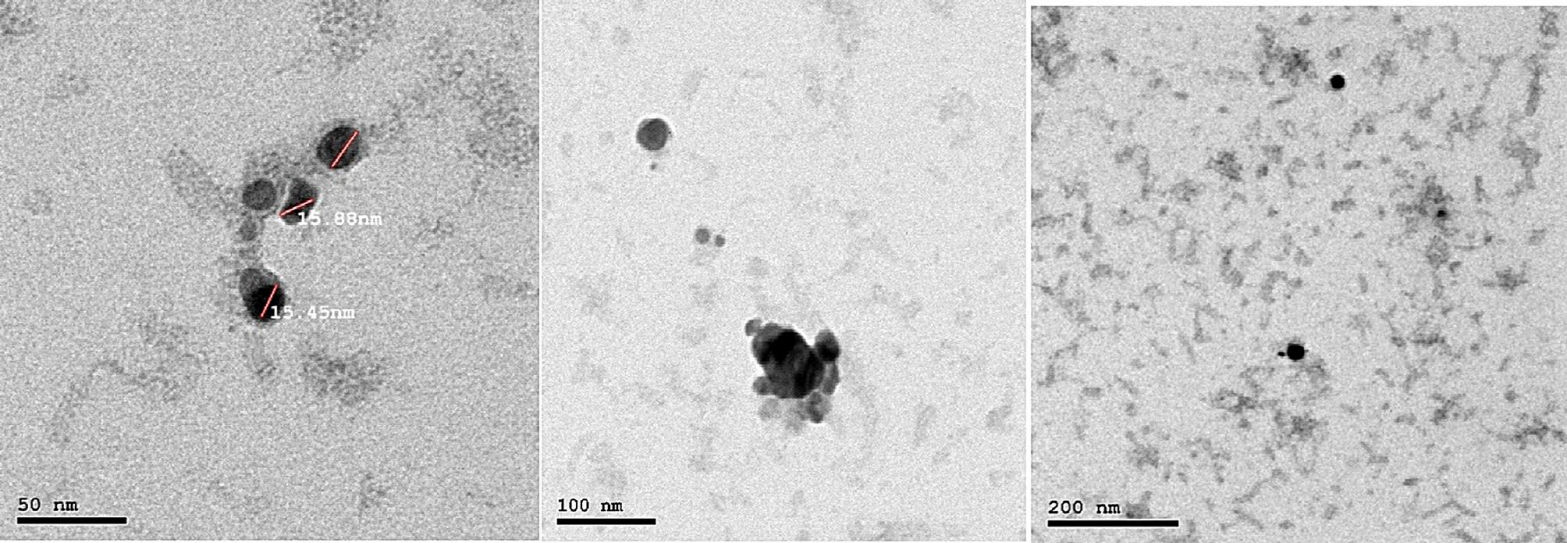
Transmission Electron Microscope (TEM) images of the prepared silver nanoparticles AgNPs at different nanoscales show a spherical shape and an average diameter of 15 nm

The average particle size assessed by the Dynamic Light scattering DLS technique was detected to be 50–500 nm for AgNPs. AgNPs stability can be simply ascertained by logging the zeta potential value. The zeta potential value registered for AgNPs was – 24.4 mV as illustrated in Fig. 2. TEM examination of AgNPs demonstrated spherical-like particles with a nanoscale range from 50 to 100 nm with a diameter of 15 nm for AgNPs as depicted in Fig. 3.

**Fig. 4:**
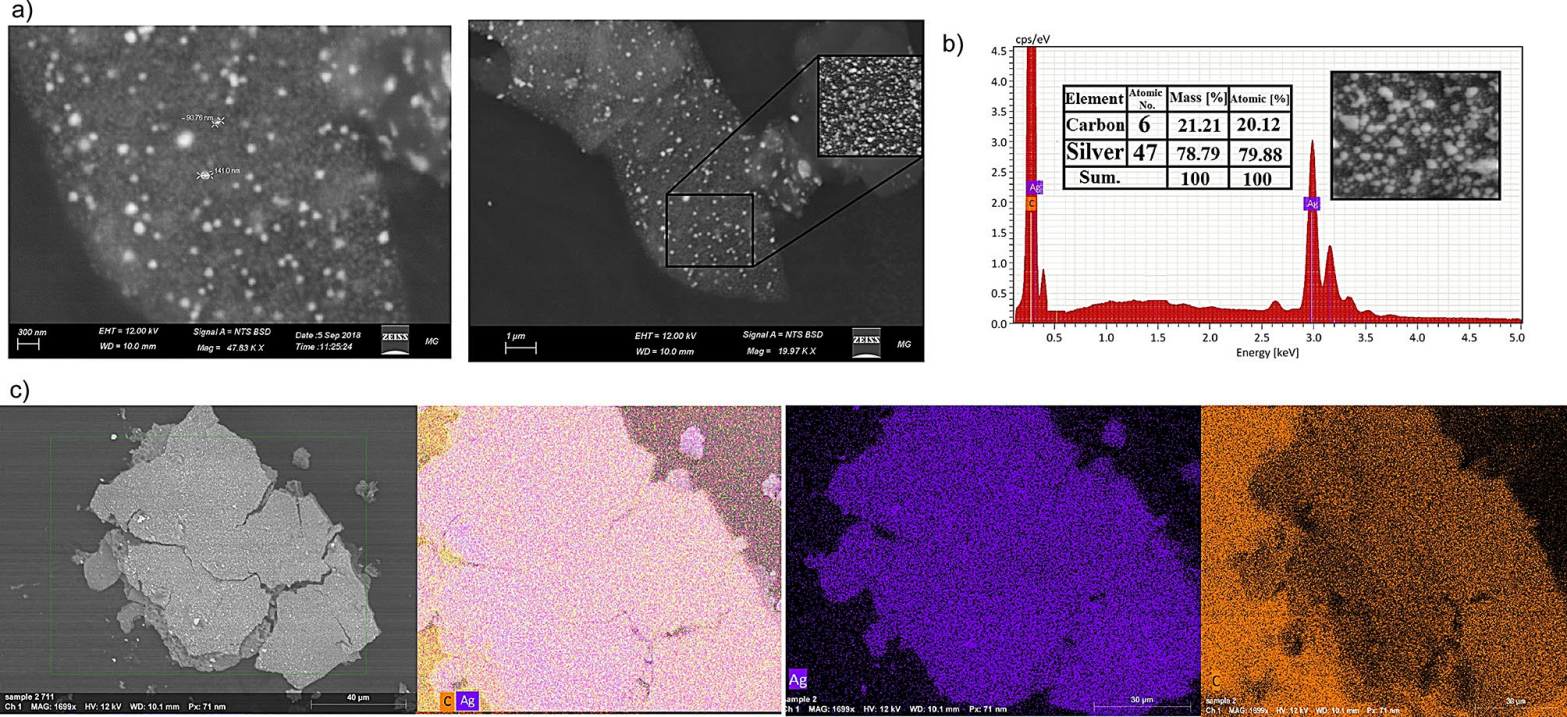
Scanning Electron Microscope (SEM), Energy-Dispersive X-ray spectra (EDX), and mapping of the prepared silver nanoparticles **(AgNPs)**: (**a**) SEM images at different magnifications showing spherical shaped particles and average diameter 96 nm and 141 nm (**b**) EDX spectrum of the prepared NPs showing silver and carbon 100% carbon and silver mass (**c**) Elemental mapping of the prepared AgNPs showing silver and carbon signals

SEM image explained the surface morphology of AgNPs which were pseudo-spherical shaped, and their size varied from 15 nm Fig. 4a. The homogeneously dispersed and well-stabilized nature of the synthesized AgNPs is also evident from the SEM pictures. The EDX mapping gives an idea about the basic examination of the synthesized NPs (Fig. 4b and c). Additional peaks seen in the EDX profile were carbon biomolecules implicated in the capping of these NPs’ surfaces, and the profile highlighted a significant signal for the presence of silver atoms.

### Bacterial isolation and identification

A total of eleven bacterial isolates were recovered from wound infections and then identified by MALDI-TOF (Supplementary Table S1). Two *Escherichia coli* isolates (EC.1 and EC.2), two *Staphylococcus lentus* isolates (St.1 and St.2), two *Klebsiella pneumonia* isolates (KP.1 and KP.2), two *Pseudomonas aeruginosa* isolates (Ps.1 and Ps.2), *Acinetobacter baumannii* (AB), *Stenotrophomonas maltophilia* (St.M) and *Staphylococcus aureus* (St.A) were identified. For MALDI-TOF MS-based bacterial ID in Clinical Microbiology Laboratories, the MALDI Biotyper system was used. (Bruker Daltonics; https://www.bruker.com/en/products-and-solutions/mass-spectrometry/maldi-tof.html).

A scoring system was used to express the pattern-matching results. A score of less than 1.7 was considered an unreliable ID, while a score of more than 2.0 was considered a species-level result. All tested bacterial isolates proved to match species level score > 2.

### MIC determination of AgNPs against the tested bacterial isolates

**Fig. 5:**
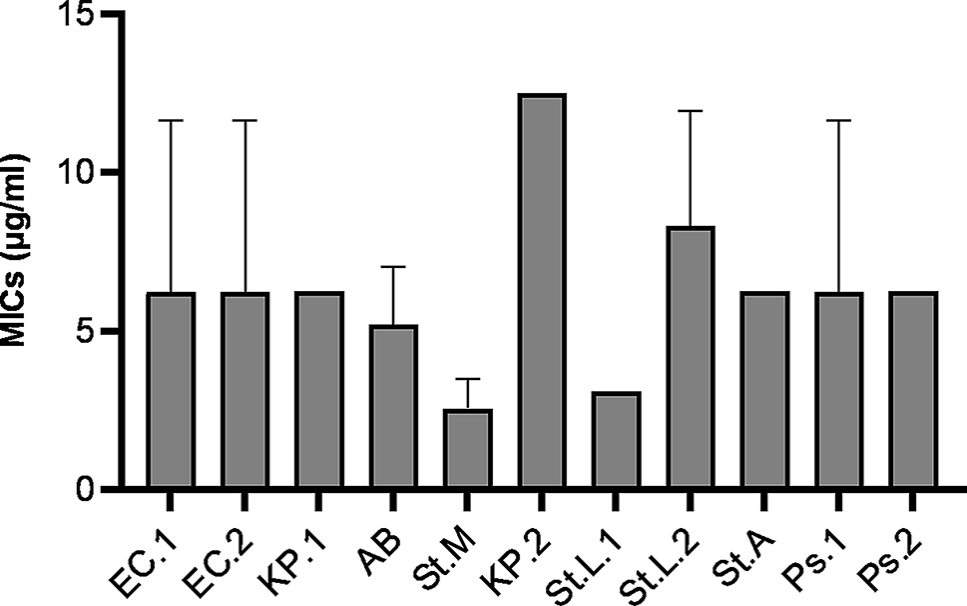
Graph showing the mean Minimum Inhibitory Concentrations (MICs) of silver nanoparticles (AgNPs) in µg/ml against the tested bacterial isolates using the Agar Dilution method. All the values are averages of triplicates and expressed as ± SD values. Abbreviations: EC.1, *Escherichia coli*; EC.2, *Escherichia coli*; KP.1, *Klebsiella pneumonia*; KP.2, *Klebsiella pneumonia*; St.L.1, *Staphylococcus lentus*; St.L.2, *Staphylococcus lentus*; St.A, *Staphylococcus aureus*; Ps.1, *Pseudomonas aeruginosa*; Ps.2, *Pseudomonas aeruginosa*; AB, *Acinetobacter baumannii; St.M*, *Stenotrophomonas maltophilia*

The antimicrobial effectiveness of AgNPs was determined by the standard methods of the CLSI 2011&2018. Discrete amounts of AgNPs were settled from 1.5 to 50 µg/ml. MIC was performed against eleven bacterial isolates by the agar dilution method. The experiment was done on three different days. The mean MIC range and standard deviation of the AgNPs fluctuated from 3.12 µg/ml to 8.3 µg/ml (Fig. 5).

### Induction and development of bacterial resistance to AgNPs

To find out if these isolates may develop resistance to AgNPs over time, they were tested against increasing concentrations of AgNPs, and the changes in bacterial sensitivity to AgNPs were determined. MICs were determined following each of the twenty consecutive steps of bacterial growth, which involved putting each tested isolate in cultivation media comprising subinhibitory doses of AgNPs (Table 1). The results clearly shows that the isolates developed resistance after the addition of AgNPs, and this resistance increased with increasing concentrations of AgNPs, stabilizing after the 20th passage. All isolates changed their susceptibility level to AgNPs to become resistant to high concentrations of AgNPs. These findings unequivocally demonstrate that following prolonged exposure every tested bacterial isolate became resistant to AgNPs. Both EC.1 & EC.2 changed sensitivity from 6.25 to > 30 µg /ml, St.L.1 & St.L.2 changed sensitivity from 3.12 µg/ml and 8.3 µg/ml to > 30 µg/ml, KP.1 & KP.2 changed sensitivity from 6.25 µg/ml and 7.2 µg/ml to > 30 µg/ml, and St. A changed susceptibility from 6.25 to > 30 µg/ml. Both Ps.1 & Ps.2 changed susceptibility from 6.25 to > 30 µg/ml and AB and St. M changed sensitivity from 5.2 µg/ml and 2.56 µg/ml to > 30 µg/ml. To verify the reasons for changing the variants’ sensitivity, whole genome sequencing was analyzed. Only two resistant isolates were chosen to be whole genome sequenced; KP.1 and St.L.1 were picked as representatives of gram-negative and gram-positive bacterial isolates, respectively, and were subjected to next-generation sequencing.

**Table 1:** Determination of Minimum Inhibitory Concentration (MIC) of silver nanoparticles (AgNPs) against tested gram-positive and gram-negative isolates after each of twenty consequent culture steps

Isolates	Mean MIC ± SD (µg/ml)	Number of passages																			
		1	2	3	4	5	6	7	8	9	10	11	12	13	14	15	16	17	18	19	20
St.L.1*	3.12	3.1	3.1	5	5	6.2	6.25	6.2	10	10	10	12.5	10	12.5	12.5	12.5	12.5	20	20	25	> 30
St.L.2	8.3 ± 3.6	6.2	6.2	10	10	10	12.5	10	12.5	12.5	12.5	20	12.5	20	12.5	20	20	20	25	20	> 30
St.A	6.25	5	5	6.2	10	6.2	6.2	10	10	10	12.5	10	12.5	12.5	12.5	20	20	20	20	25	> 30
KP.2	7.2	6.2	10	10	12.5	10	12.5	12.5	12.5	20	12.5	20	12.5	20	25	20	25	20	25	25	> 30
KP.1*	6.25	5	5	10	6.2	10	6.2	10	10	10	20	20	20	20	25	20	20	25	25	30	> 30
Ps.1	6.25 ± 5.4	5	6.2	10	6.2	6.2	6.2	10	10	10	12.5	10	12.5	12.5	12.5	12.5	20	12.5	20	20	> 30
Ps.2	6.25 ± 5.4	5	5	6.2	10	12.5	10	12.5	12.5	12.5	20	12.5	12.5	20	20	25	20	20	25	25	> 30
EC.1	6.25 ± 5.4	5	5	6.2	6.2	10	10	10	12.5	10	10	12.5	12.5	12.5	12.5	20	20	25	25	25	> 30
EC.2	6.25 ± 5.4	5	5	6.2	6.2	10	10	10	10	12.5	10	12.5	12.5	12.5	20	12.5	20	20	20	25	> 30
AB	5.2 ± 1.8	3.1	5	5	6.2	6.25	6.2	10	10	10	12.5	10	12.5	12.5	12.5	20	12.5	20	20	25	> 30
St. M	2.56 ± 0.9	1.5	3.1	3.2	6.2	6.25	10	10	12.5	12.5	12.5	12.5	20	12.5	12.5	20	20	20	25	25	> 30

*Bacterial isolates selected for whole genome sequence

### Genome sequencing, assembly, and annotation

The sequenced data were subjected to contamination screening and the genome sizes of final assemblies were 5,785,228 Kbp for KP.1 isolate and 2,863,362 Kbp for St.L.1 isolate. The de novo assembly of genome sequence data revealed that the number of contigs (> 200 bp) was 253 for KP.1 and 148 for St.L.1 isolate. More than 80% of the reads are above the Phred quality score of 30 indicating high-quality sequencing data. These contigs were aligned to the reference genome of each isolate. The Maximum contig sizes were 250,107 bp and 245,449 bp for KP.1 and St.L.1 respectively. The GC content was 56.58% for KP.1 and 32.63% for St.L.1. A summary of the genome sequence data and assembly are shown in Table 2.

**Table 2:** Genomic annotation analysis of the control isolates *Klebsiella pneumonia* (KP.1) and *Staphylococcus lentus* (St.L.1)

Strain	Number of contigs	Reads	Genome size (bp)	Maximum contig size (bp)	Minimum contig size (bp)	N50 size (bp)	% of *≥* Q30 bases	GC Content (%)	Completeness (%)
KP.1	253	391,924	5,785,228	250,107	300	82,529	30	56.58	100
St.L.1	148	1,603,261	2,863,362	245,449	300	73,196	30	32.63	99.4

### Genome single nucleotide polymorphism

**Fig. 6:**
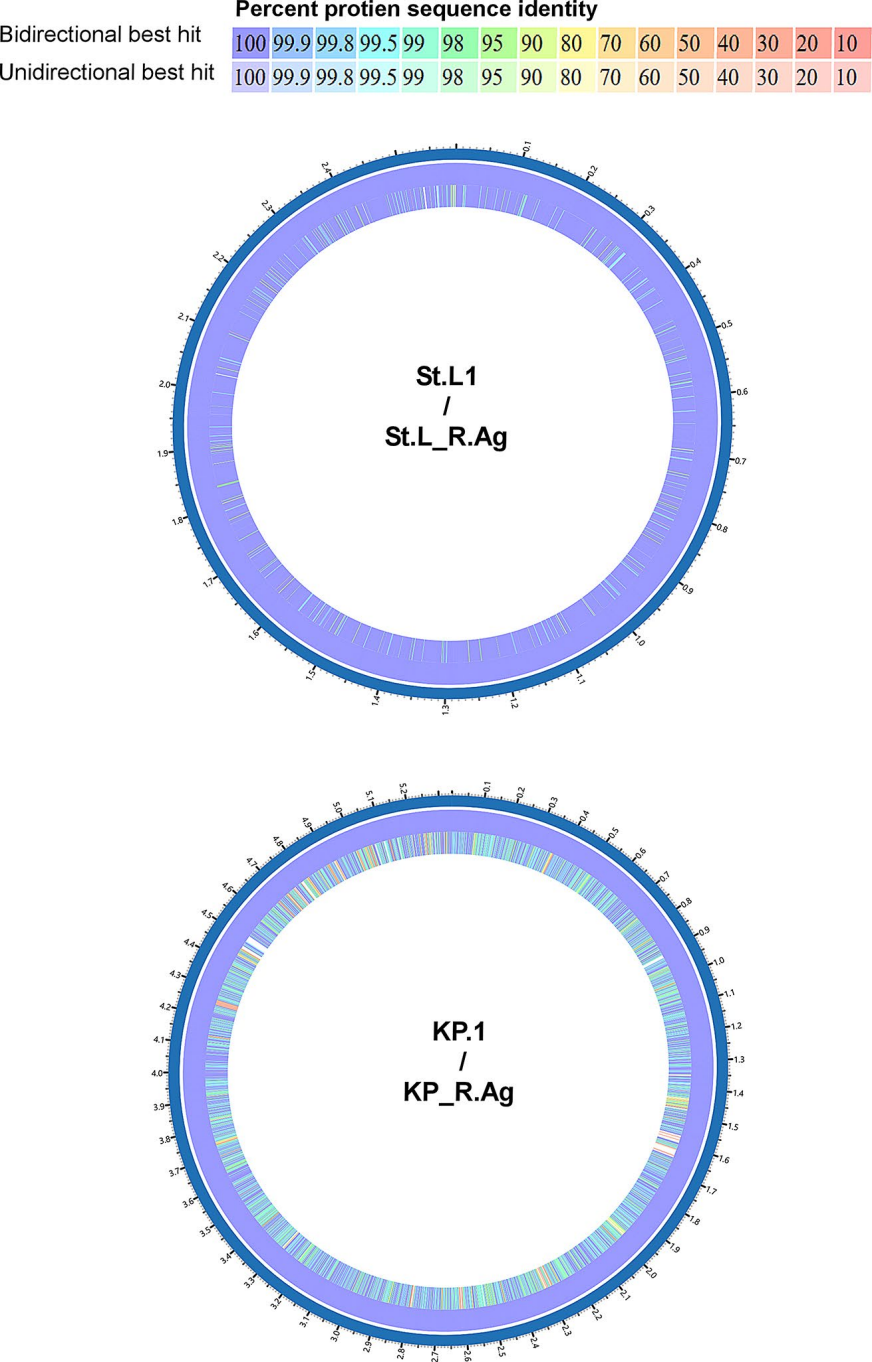
Circular genomic map of silver nanoparticles (AgNPs) resistant isolates *Staphylococcus lentus* (St.L_R.Ag) and *Klebsiella pneumonia* (KP_R.Ag) compared to their reference genomes St.L.1 and KP.1, respectively. The innermost circle represents the genomes of the AgNPs resistant isolates, while the outermost circle represents the genomes of their corresponding control isolates. Colors indicate BLASTP matches from 10–100% nucleotide identities. Blank regions denote non-coding areas

Figure 6 shows the circular map of the two AgNPs resistant isolates KP_R.Ag and St.L_R.Ag compared to the reference genome KP.1 and St.L.1 respectively. The inner circle represents the genome of the resistant AgNPs isolate, while the outer circle represents the genome of their corresponding control isolate. The shared identity of each isolate with the reference genome is represented in different colors, which denotes the BLASTP matches between 10 and 100% nucleotide identities. The blank regions in the rings represent the areas of non-coding regions, unannotated sequences, or regions with insufficient data coverage.

**Fig. 7:**
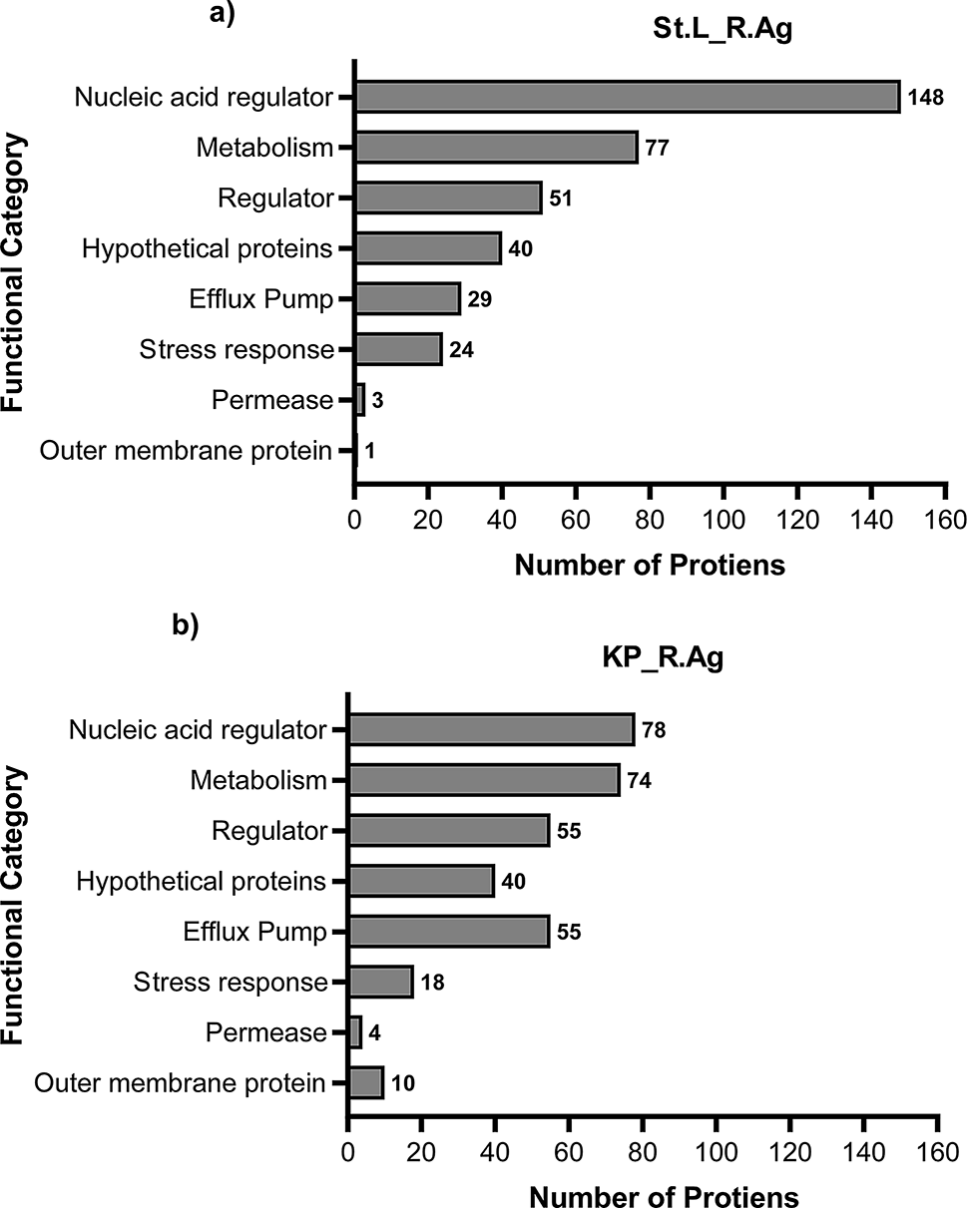
Functional categories of enzymes/proteins affected by mutations in bacterial genomes: **a**) Number of proteins in *Staphylococcus lentus* (St.L_R.Ag) resistant isolate **b**) Number of proteins in *Klebsiella pneumonia* (KP_R.Ag) isolate

We focused our analysis on identifying nonsense SNPs that result in premature termination of functioning genes. In addition, we also screened for point mutations that lead to the change of codons or nucleotide deletions or insertions that create frameshift mutations. In resistant isolates, we first looked for nsSNPs in comparison to their reference genome. nsSNPs were identified in KP_R.Ag and St.L_R.Ag isolates compared to their reference isolate. To probe nsSNPs in genes annotated to be associated with AgNPs resistance [36] or virulence factors [37], we selectively extracted the nsSNPs that existed in divergent NPs resistant genes of resistant isolates. Many of the polymorphic genes among the resistant isolates were correlated with virulence, metabolism, and Nucleic acid regulator genes involved in the repair process. In the St.L_R.Ag isolate (Fig. 7a), our analysis identified 40 nsSNPs coding for hypothetical proteins, 29 nsSNPs coding for efflux pump proteins, and 51, 77, and 148 nsSNPs coding for regulators, metabolism-related proteins, and nucleic acid regulators, respectively. Additionally, among the genes linked to AgNPs resistance, 24 stress response genes, 3 permease proteins, and 1 outer membrane protein were discovered.

The number of nsSNPs shared within a gene across KP_R.Ag isolates are depicted in Fig. 7b. For instance, KP_R.Ag isolate that codes for hypothetical proteins included 40 nsSNPs. AgNPs efflux pump proteins are coded for by 55 nsSNPs; nucleic acid regulators, metabolism, and regulators are coded for by 78, 74, and 55, respectively. Additionally, we also found out nsSNPs in genes responsible for resistance to AgNPs, including four permeases, ten outer membrane proteins, and 18 stress response genes. Collectively, these were discovered to influence cell wall permeability and contribute to nanoparticle resistance [38].

Focusing on permeases, outer membrane proteins, and efflux pumps, as shown in Table 3, the KP_R.Ag isolate possesses 26 nucleotide deletions, causing a frameshift change that truncates the protein products. Also, it was detected that 13 nucleotide insertions led to frameshift mutations, 5 nonsynonymous variations caused stop codon loss, and 26 nonsynonymous variations resulted in missense variants with moderate SNP impact. On the other hand, as described in Table 4, St.L_R.Ag possesses 1 nucleotide deletion with high SNP impact provoking a frameshift change and 31 nonsynonymous variants that lead to missense moderate impact. The thorough list of unique nsSNPs is marked in KP_R.Ag and St.L_R.Ag compared to AgNPs susceptible isolates is shown in Supplementary Tables S2 & S3.

**Table 3:** Key nsSNPs identified in *Klebsiella pneumonia* (KP_R.Ag) resistant isolate compared to KP.1 reference genome

SNP type	Reference Nucleotide	Variant Nucleotide	Reference amino acid position change	SNP effect type	SNP effect impact	Protein /enzyme	Function
Deletion	Ggccga	GGCGa	Gly282fs	Frameshift variant	High	Permease of the drug/metabolite transporter (DMT) superfamily	Permease
Non-synonymous	Taa	Caa	Ter201Glnext*?	Stop Lost	High	Inner membrane protein **YbiR**, putative anion permease	Permease
Deletion	Acgttttttattagc	acGTTTTTATTAGc	Phe210fs	Frameshift variant	High	Cytosine/purine/uracil/thiamine/allantoin permease family protein	Permease
Deletion	actttttttcgc	aCTTTTTTCGC	Phe246fs	Frameshift variant	High	Fucose	Permease
Non-synonymous	Acc	aAc	Thr75Asn	Missense variant	Moderate	Outer membrane protein X precursor	Outer membrane protein
Non-synonymous	Gtgctg	gCCCTG	Val118Ala	Missense variant	Moderate	Outer membrane porin **OmpN**	Outer membrane protein
Non-synonymous	Gaa	Aaa	Glu16Lys	Missense variant	Moderate	Outer membrane low permeability porin, **OprD**	Outer membrane protein
Non-synonymous	Aacaat	aaCGAT	Asn240Asp	Missense variant	Moderate	Outer membrane porin **PhoE**	Outer membrane protein
Insertion	Ccctcgccctgctgcaag	CTCAAGGTGATCCTCGccctgctgcaag	Pro151_Ser152fs	Frameshift variant	High	Fimbrial adhesion	Outer membrane protein
Deletion	Cggcaaaaaaaacgg	cGGCAAAAAAACgg	Gln494fs	Frameshift variant	High	**FimD** Outer membrane usher protein	Outer membrane protein
Deletion	Ggcgacgacacc	ggCGACAcc	Gly134_Asp135del	Missense variant	Moderate	Outer membrane porin **OmpC**	Outer membrane protein
Non-synonymous	Agc	Cgc	Ser626Arg	Missense variant	Moderate	Outer membrane protein assembly factor **YaeT**	Outer membrane protein
Deletion	Cgggataaaaaacgt	cgGGATAAAAACgt	Lys298fs	Frameshift variant	High	Outer membrane protein/protective antigen	Outer membrane protein
Insertion	Gaccgctgc	gACCCGCTgc	Arg311_Cys312fs	Frameshift variant	High	Carbohydrate-selective porin **OprB**	Outer membrane protein
Insertion	Attgccgcg	atTGCCCGCg	Ala153_Ala154fs	Frameshift variant	High	ABC transporter ATP binding protein	Efflux pump
Insertion	Ggcgcc	ggCGACc	Ala177_Arg178fs	Frameshift variant	High	ABC transporter, substrate-binding protein (cluster 1, maltose/g3p/polyamine/iron)	Efflux pump
Insertion	Cgcgct	cGCCCGct	Ala130_Asp131fs	Frameshift variant	High	Iron compound ABC transporter, permease protein	Efflux pump
Insertion	Agcgct	AGCGCGct	Ala163_Ala164fs	Frameshift variant	High	ABC transporter, substrate-binding protein	Efflux pump
Insertion	Atgcgc	ATTGcgc	Met48_Arg49fs	Frameshift variant	High	ABC transporter involved in cytochrome c biogenesis, **CcmB** subunit	Efflux pump
Non-synonymous	Tga	tCa	Ter96Serext*?	Stop lost	High	Phospholipid ABC transporter-binding protein **MlaB**	Efflux pump
Deletion	gagaaaaaaagcggc	gaGAAAAAAGCGgc	Ser251fs	Frameshift variant	High	Maltodextrin ABC transporter, substrate-binding protein **MdxE**	Efflux pump
Deletion	Ggcggttttttttgc	ggCGGTTTTTTTGc	Gly60fs	Frameshift variant	High	Branched-chain amino acid ABC transporter, ATP-binding protein **LivF**	Efflux pump
Deletion	ctgccgccggcggac	ctGCGCCGGCGGAC	Pro164fs	Frameshift variant	High	Alkanesulfonate ABC transporter substrate-binding protein **SsuA**	Efflux pump
Deletion	Ctgttttttttgttcgcg	ctGTTTTTTTGTTCGCg	Leu6fs	Frameshift variant	High	Zinc ABC transporter, substrate-binding protein **ZnuA**	Efflux pump
Deletion	Gctaaaaaaagt	GCTAAAAAAGt	Lys128fs	Frameshift variant	High	ABC transporter, ATP-binding protein (cluster 3, basic aa/glutamine/opines)	Efflux pump
Deletion	Acggct	aCGCT	Ala56fs	Frameshift variant	High	ABC-type phosphate/phosphonate transport system, periplasmic component	Efflux pump
Deletion	Gagaaaaaaaatgaatat	gaGAAAAAAATGAATAT	Asn77fs	Frameshift variant	High	Glutamate/aspartate ABC transporter, substrate-binding protein **GltI**	Efflux pump
Deletion	Actttttttcgc	aCTTTTTTCGC	Phe191fs	Frameshift variant	High	ABC transporter, permease protein 2 (cluster 5, nickel/peptides/opines)	Efflux pump
Non-synonymous	Tga	Cga	Ter335Argext*?	Stop Lost	High	ABC-type Fe3+-siderophore transport system, permease component	Efflux pump
Deletion	Acctttttttggccg	acCTTTTTTGGCcg	Trp237fs	Frameshift variant	High	**YehX** Osmoprotectrant ABC Transporter	Efflux pump
Insertion	acgttttttcttttttcgttg	AcGTTTTTTTCTTTTTTCGttg	Leu38_Phe39fs	Frameshift variant	High	Ferrous iron transporter-associated protein **FeoA**	Efflux pump
Non-synonymous	Acg	aGg	Thr331Arg	Missense variant	Moderate	Efflux ABC transporter, permease/ATP-binding protein **MdlB**	Efflux pump
Deletion	Ctgtttttaccccgcggggtgatg	ctGTTTTTACCCCGCGGGTGAtg	Val348fs	Frameshift variant	High	Urea ABC transporter **UrtC**	Efflux pump
Insertion	Tcgccg	tCGGCcg	Pro246_Ser247fs	Frameshift variant	High	Methionine ABC transporter substrate-binding protein	Efflux pump
Insertion	Gacgtc	gACCGtc	Val454_Ser455fs	Frameshift variant	High	Maltodextrin ABC transporter, permease protein **MdxF**	Efflux pump
Deletion	Caggtctttttttaa	caGGTCTTTTTTAa	Phe202fs	Frameshift variant	High	N-Acetyl-D-glucosamine ABC transport system, permease protein	Efflux pump
Deletion	Tacaagaaagaaaaaaaacgt	TACAAGAAAGAAAAAAACgt	Glu51fs	Frameshift variant	High	Molybdenum ABC transporter, substrate-binding protein **ModA**	Efflux pump
Non-synonymous	Cagttt	ATGTTC	GlnPhe156MetPhe	Missense Variant	Moderate	Oligopeptide ABC transporter, ATP-binding protein **OppF**	Efflux pump
Non-synonymous	Tca	GCG	Ser30Ala	Missense variant	Moderate	Bacteriocin/lantibiotic efflux ABC transporter, permease ATP-binding protein	Efflux pump
Deletion	Gccaaa	GCAAa	Ala212fs	Frameshift variant	High	Inner-membrane proton/drug antiporter (MSF type) of tripartite multidrug efflux system	Efflux pump
Non-synonymous	Gtc	gCc	Val262Ala	Missense variant	Moderate	Multidrug efflux pump **MdtL** (MFS type)	Efflux pump
Deletion	Gtttttccg	GTTTTCCG	Phe232fs	Frameshift variant	High	Uncharacterized MFS-type transporter	Efflux pump
Insertion	Gccgcc	gcCGGCc	Ala264_Gly265fs	Frameshift variant	High	Multidrug efflux pump **EmrD** (MFS type)	Efflux pump
Deletion	Ctttttttatcc	CTTTTTTATCC	Leu330fs	Frameshift variant	High	3-(3-hydroxyphenyl) propionate transporter, MFS-type	Efflux pump
Non-synonymous	Att	aGt	Ile104Ser	Missense variant	Moderate	Multidrug efflux pump **MdtM** (MFS type)	Efflux pump
Non-synonymous	Ctg	ATC	Leu959Ile	Missense variant	Moderate	Multidrug efflux system AcrAB-TolC, inner-membrane proton/drug antiporter AcrB (RND type)	Efflux pump
Non-synonymous	ggcaccaccggcgcc	ggCTCAACCGGCGCc	Thr462Ser	Missense variant	Moderate	Aminoglycosides efflux system AcrAD-TolC, inner-membrane proton/drug antiporter AcrD (RND type)	Efflux pump
Non-synonymous	Gatgaaaac	gaTAAAAAC	Glu639Lys	Missense variant	Moderate	Multidrug efflux system AcrEF-TolC, inner-membrane proton/drug antiporter AcrF (RND type)	Efflux pump
Non-synonymous	Tactga	tGCTTa	TyrTer203CysLeuext*?	Stop lost	High	RND efflux system, membrane fusion protein	Efflux pump
Deletion	Gtcaaaaaaaac	GTCAAAAAAAC	Lys132fs	Frameshift variant	High	Multidrug efflux system EmrAB-OMF, membrane fusion component EmrA	Efflux pump
Deletion	Aattttttttccgtc	aATTTTTTTCCGtc	Ser554fs	Frameshift variant	High	Putative heavy metal/multi-drug efflux protein, RND family	Efflux pump
Non-synonymous	Tat	tTt	Tyr166Phe	Missense variant	Moderate	Copper/silver efflux RND transporter, transmembrane protein **CusA**	Efflux pump
Non-synonymous	Agtgct	CATGCC	SerAla81HisAla	Missense variant	Moderate	Copper/silver efflux RND transporter, membrane fusion protein **CusB**	Efflux pump
Non-synonymous	Ccg	Acg	Pro23Thr	Missense variant	Moderate	Copper/silver efflux RND transporter, outer membrane protein **CusC**	Efflux pump
Non-synonymous	Acg	aTT	Thr367Ile	Missense variant	Moderate	Copper sensory histidine kinase **CusS**	Efflux pump
Non-synonymous	Ccc	Tcc	Pro7Ser	Missense variant	Moderate	Inner membrane component of tripartite multidrug resistance system **MdtN**	Efflux pump
Non-synonymous	Cta	cCa	Leu199Pro	Missense variant	Moderate	Membrane fusion component of tripartite multidrug resistance system **MdtO**	Efflux pump
Insertion	Tgggggggcttg	TGGGGGGGGCTTg	Gly74_Leu75fs	Frameshift variant	High	Multidrug resistance protein **MdtG**	Efflux pump
Non-synonymous	Ctg	cAg	Leu21Gln	Missense Variant	Moderate	Multidrug resistance outer membrane protein **MdtP**	Efflux pump
Non-synonymous	Cga	cAa	Arg73Gln	Missense variant	Moderate	Putative multidrug resistance outer membrane protein **MdtQ**	Efflux pump
Deletion	Actttttttgtttggcgc	aCTTTTTTGTTTGGCgc	Phe114fs	Frameshift variant	High	Inner membrane transporter **YjeM**	Efflux pump
Deletion	Ccgttttgcataaaaaaaagc	cCGTTTTGCATAAAAAAAGc	Ile25fs	Frameshift variant	High	Mg/Co/Ni transporter **MgtE**, CBS domain-containing	Efflux pump
Deletion	Ccggattttttttat	ccGGATTTTTTTAt	Asp46fs	Frameshift variant	High	**KefG** Glutathione regulated potassium efflux system ancillary protein	Efflux pump
Non-synonymous	Tcc	Acc	Ser387Thr	Missense variant	Moderate	Efflux transport system, outer membrane factor (OMF) lipoprotein	Efflux pump
Non-synonymous	Att	GTC	Ile32Val	Missense variant	Moderate	Nickel/cobalt efflux system	Efflux pump
Insertion	Gcacca	GCCAcca	Ala314_Pro315fs	Frameshift variant	High	2-nitroimidazole transporter **NimT**	Efflux pump
Non-synonymous	Atg	atA	Met1?	Start lost	High	**YnfA** protein	Efflux pump
Non-synonymous	Atcggattc	atTGGCAtc	IleGlyPhe95IleGlyIle	Missense variant	Moderate	Small multidrug resistance (SMR) efflux transporter	Efflux pump
Non-synonymous	Gtc	gCc	Val224Ala	Missense variant	Moderate	Multidrug efflux transporter **MdtK/NorM** (MATE family)	Efflux pump

**Table 4:** Key nsSNPs identified in the *Staphylococcus lentus* (St.L_R.Ag) resistant isolate compared to St.L.1 reference genome

SNP type	Reference Nucleotide	Variant Nucleotide	Reference amino acid position change	SNP effect Impact	SNP effect Type	Protein /enzyme	Function
Non-synonymous	Atg	AAg	Met147Lys	Moderate	Missense variant	Macrolide export ATP binding/ permease protein	Permease
Non-synonymous	Caaaatatc	caTAATTTA	GlnAsnIle575HisAsnLeu	Moderate	Missense variant	Uncharacterized amino acid permease **YdaO**	Permease
Non-synonymous	Tta	ttT	Leu149Phe	Moderate	Missense variant	Amino-acid permease **AapA**	Permease
Non-synonymous	Aga	Aag	Arg113Lys	Moderate	Missense variant	Spermidine/putrescine import ABC transporter ATP-binding protein **PotA**	Efflux pump
Non-synonymous	Acagcaaaa	acGGCACaa	ThrAlaLys271ThrAlaGln	Moderate	Missense variant	Spermidine/putrescine import ABC transporter substrate-binding protein **PotD**	Efflux pump
Non-synonymous	Gcattt	gcTTTA	AlaPhe194AlaLeu	Moderate	Missense variant	Spermidine/putrescine import ABC transporter permease protein **PotC**	Efflux pump
Non-synonymous	Gtc	Atc	Val260Ile	Moderate	Missense variant	Excinuclease ABC subunit C	Efflux pump
Non-synonymous	Ggg	AGT	Gly80Ser	Moderate	Missense variant	Excinuclease ABC subunit B	Efflux pump
Non-synonymous	Acatta	acTAta	ThrLeu46ThrIle	Moderate	Missense variant	Excinuclease ABC subunit A	Efflux pump
Non-synonymous	Ggc	CGT	Gly200Arg	Moderate	Missense variant	ABC transporter, ATP-binding protein (cluster 3, basic aa)	Efflux pump
Non-synonymous	Aaa	TTa	Lys45Leu	Moderate	Missense variant	ABC transporter, permease protein	Efflux pump
Non-synonymous	Caa	caT	Gln192His	Moderate	Missense variant	ABC transporter, ATP-binding protein (cluster 15, trp?	Efflux pump
Non-synonymous	Gac	gaA	Asp96Glu	Moderate	Missense variant	Maltodextrin ABC transporter, ATP-binding protein **MsmX**	Efflux pump
Non-synonymous	Caa	cTa	Gln371Leu	Moderate	Missense variant	Maltodextrin ABC transporter, substrate-binding protein **MdxE**	Efflux pump
Non-synonymous	Gtg	gCT	Val162Ala	Moderate	Missense variant	Phosphate ABC transporter, ATP-binding protein **PstB**	Efflux pump
Non-synonymous	Gtggtt	gtTTtt	ValVal91ValPhe	Moderate	Missense variant	ABC transporter, ATP-binding protein (cluster1/maltose/g3p/polyamine/iron)	Efflux pump
Non-synonymous	Gga	gCa	Gly206Ala	Moderate	Missense variant	Glycine betaine ABC transport system ATP-binding protein **OpuA**	Efflux pump
Non-synonymous	Ccgact	ccTTct	ProThr384ProSer	Moderate	Missense variant	Heterodimeric efflux ABC transporter, Multidrug resistance	Efflux pump
Non-synonymous	Act	aAt	Thr422Asn	Moderate	Missense variant	ABC transporter, Permease /ATP-binding protein	Efflux pump
Deletion	Tatcct	tACct	Tyr142fs	High	Frameshift variant	ABC transporter-like sensor ATP-binding protein	Efflux pump
Non-synonymous	Ggctat	ggTTTt	GlyTyr40GlyPhe	Moderate	Missense variant	Methionine ABC transporter ATP-binding protein	Efflux pump
Non-synonymous	Gccaat	gcAAGt	AlaAsn520AlaSer	Moderate	Missense variant	Efflux ABC transporter, permease/ATP-binding protein **MdlB**	Efflux pump
Non-synonymous	Gat	gaA	Asp155Glu	Moderate	Missense variant	Iron compound ABC uptake transporter ATP-binding protein	Efflux pump
Non-synonymous	Gat	gaA	Asp18Glu	Moderate	Missense variant	Bis-ABC ATPase **YbiT**	Efflux pump
Non-synonymous	Gtaggaaca	gtTGGTCAa	ValGlyThr68ValGlyGln	Moderate	Missense variant	Manganese ABC transporter, periplasmic-binding protein **SitA**	Efflux pump
Non-synonymous	Ttaggggtt	ATTGGAAtt	LeuGlyVal68IleGlyIle	Moderate	Missense variant	Oligopeptide ABC transporter, ATP-binding protein **OppF**	Efflux pump
Non-synonymous	Ttgaac	ttACac	LeuAsn279LeuHis	Moderate	Missense variant	Oligopeptide ABC transporter, ATP-binding protein **OppD**	Efflux pump
Non-synonymous	Caa	Gaa	Gln235Glu	Moderate	Missense variant	Na + dependent nucleoside transporter **NupC**	Efflux pump
Non-synonymous	Agt	aAC	Ser10Asn	Moderate	Missense variant	Na (+)-dependent branched-chain amino acid transporter	Efflux pump
Non-synonymous	Att	Gtt	Ile63Val	Moderate	Missense variant	Di-tripeptide/cation symporter **DtpT**	Efflux pump
Non-synonymous	Agt	agG	Ser32Arg	Moderate	Missense variant	Cation-transporting ATPase, E1-E2 family	Efflux pump
Non-synonymous	Tta	Ata	Leu93Ile	Moderate	Missense variant	Substrate-specific component of predicted queuosine-regulated ECF transporter **QueT**	Efflux pump

Further examination unveiled the presence of mutations within the Adenosine triphosphate binding cassette (ABC) transport system encompassing different groups of proteins in KP_R.Ag isolate. Among these, mutations in the ATP binding proteins were identified in LivF, MdlB, OppF, Bacteriocin/Lantibiotic efflux along with clusters related to basic amino acids, glutamines, and opines while alterations in the substrate-binding proteins were observed in, ModA, MdxE, SsuA, ZnuA, GltI, Methionine, phosphate/phosphonate periplasmic component and clusters related to maltose, polyamine and iron. Additionally, mutations affecting the permease proteins were found in MdxF, N-acetyl-D-glucosamine, iron compound, and F^+ 3^ siderophore transport system along with clusters related to nickel, peptides, and opines. Furthermore, mutations were detected in proteins involved in specialized transport functions, such as FeoA for ferrous iron transport, YehX for osmoprotectant transport, UrtC for urea transport, and MlaB for phospholipid binding protein transport. These mutations collectively highlight the diverse genetic changes within the ABC transport system, potentially influencing various cellular transport processes. As for the St.L_R.Ag isolate, mutations were detected in genes of PotA, PotD, PotC, YbiT, OpuA, PstB, and MsmX, along with clusters related to maltose, basic amino acids, and iron.

Within the KP_R.Ag isolate, we identified mutations in various multidrug efflux transporter proteins belonging to the RND family such as CusA, CusB, CusC, AcrAB-TolC, AcrAD-TolC, AcrEF-TolC, putative heavy metals, and membrane fusion protein. These mutations are integral to the function of the multidrug efflux pump system, enhancing the KP_R.Ag isolate’s resistance mechanism. Additionally, mutations were detected in outer membrane proteins/porins, including OmpN, OprD, PhoE, OmpC, YaeT, OprB, FimD, outer membrane protein X, and outer membrane protective antigen. These mutations play a crucial role in altering membrane permeability, thereby contributing to the KP_R.Ag isolate’s enhanced resistance capabilities.

Furthermore, the St.L_R.Ag isolate exhibited the presence of mutations within the Adenosine triphosphate binding cassette (ABC) transport system. Mutations detected in the ATP binding proteins were PotA, MsmX, PstB, OpuA, MdlB, OppF, OppD, Methionine, along with clusters related to basic amino acids, maltose, polyamine, iron, while alterations in the substrate-binding proteins were observed in, PotD, MdxE, SitA.

Several stress response proteins and genes, indicative of SOS response-induced chromosomal mutations, were also identified in both the KP_R.Ag and St.L_R.Ag isolates. In the KP_R.Ag isolate, mutations were detected in genes such as DegQ, DegS, YdcZ, HSP, GrpE, YdaA, YciE, RecF, KatE, ZraP, CopD, CueR, RcsA coregulatory RcsB, superoxide dismutase protein, and universal stress response protein G (Supplementary Table S2). Conversely, the St.L_R.Ag isolate exhibited mutations in genes including RecA, LexA, CSP, HSP, TrxB, DnaK, DnaJ, ClpB, ClpP, ClpC, ClpX, ClpE, PhoH, YicC, FtsH, GlcU, AhpC, AhpF, HslO, adenylate kinase, arsenate reductase, superoxide dismutase, and MBL-fold metallohydrolase superfamily (Supplementary Table S3). In addition, mutations were detected in genes associated with antibiotic resistance in the KP_R.Ag isolate, including albicidin, fosmidomycin, and class A beta-lactamase. Conversely, mutations were found in genes encoding tetracycline and macrolide efflux proteins in the St.L_R.Ag isolate, indicating potential alterations in antibiotic resistance mechanisms.

## Discussion

As NPs are relatively new, the risk should be properly evaluated when used as antimicrobial agents. To verify the unauthorized use of NPs, strict global regulations should be developed and followed. Strict norms and regulations are in place for the use, production, and disposal of nanowaste. Much like with antibiotics, exposing organisms over time to sub-lethal dosages of NPs may hasten the evolution of resistant bacteria [21]. Sub-lethal dosages of AgNPs have been linked to multiple studies showing genetic abnormalities, changes, and overproduction of proteins/enzymes [39, 40]. It is necessary to strictly adhere to the guidelines for the concentrations of NPs used in diverse circumstances.

In this work, in order to identify the genes involved in AgNPs resistance, resistance was induced in different bacterial isolates. The chemical reduction process was used to create AgNPs. The addition of silver nitrate solution to sodium borohydride caused the solution color to shift to dark brown, signifying the formation of AgNPs [41]. After exposure to gamma irradiation at 5 kGy and 10 kGy, the remaining Ag^+^ ions after chemical preparation were reduced to Agº giving more stable NPs in the solutions. UV-visible spectrophotometer and FTIR were performed before and after irradiation to confirm AgNPs stabilization. The UV-visible spectrophotometer was adopted to analyze the formation and stability of the AgNPs in the solution. The maximum absorbance peaks for AgNPs were 0.8 and 400 nm in wavelength for control. After exposure to gamma irradiation, the absorbance increased to 1.7 and 1.9 at 5 kGy and 10 kGy, respectively. This property is called the Surface Plasmon Resonance (SPR) of AgNPs [42]. High SPR of UV analysis after exposure to gamma irradiation indicates increased concentration of AgNPs. FTIR confirms the complete reduction of all Ag^+^ ions to Agº after gamma irradiation by the effect of radiolysis. The prepared AgNPs were spherical in shape with an average size of 50–500 nm and zeta potential (-24.4) indicating highly stable AgNPs. Increased particle size in DLS analysis than in TEM analysis is due to the hydrodynamic radius of water molecules around the prepared AgNPs in solution. The SEM, EDX, and mapping of AgNPs were used to analyze the morphological structure of the NPs [43].

Following the exposure of bacterial isolates to increasing concentrations of AgNPs, the changes in bacterial susceptibility to AgNPs were demonstrated. Meanwhile, there is no standard procedure to compare the activity of AgNPs on different strains of bacteria, as the physicochemical characteristics of the nanoparticle, the bacterial growth medium, and even the incubation conditions used, would influence its activity [44–46].

Focusing on one-gram negative bacteria (KP.1) and one-gram positive bacteria (St.L.1), MIC was increased from 6.25 to > 30 µg/ml and from 3.12 to > 30 µg/ml, respectively under the same conditions. Only KP.1, St.L.1, and their AgNPs resistant isolates (KP_R.Ag and St.L_R.Ag) were chosen to be whole genome sequenced. Our results are in agreement with Panáček et al. 2018 where they demonstrated the resistance of the Gram-negative isolates *Escherichia coli* and *Pseudomonas aeruginosa* to AgNPs after repeated exposures. They determined the MIC of AgNPs and their results focused on bacteria repeatedly exposed to sub-inhibitory concentrations of AgNPs, where pathogens were able to rapidly develop AMR [47].

In this case, we have looked into the genetic variety in *Klebsiella pneumonia* and *Staphylococcus lentus* hospital wound isolates by next-generation sequencing NGS and contrasting genomics. Utilizing the WGS technique to evaluate the isolates’ genome sequences (AgNPs-resistant) and contrasting the results with the reference genome allowed researchers to explore the genomic variance among the isolates with different resistance traits (AgNPs-susceptible).

Many nsSNPs are potentially significant in AMR, with new perspectives to expand our knowledge of the aspects influencing the hospital isolates’ resistance regime. Multiple processes of antimicrobial resistance in both *Klebsiella pneumonia* and *Staphylococcus lentus* isolates have been identified [48–50]. Mechanisms of antimicrobial resistance entail the following: changes in the antimicrobial agent target to alter the antimicrobial’s affinity, antimicrobial deactivation via synthetic alteration or deterioration, alterations in antimicrobial agent permeability via modifications affecting the bacterial cell surface (e.g., by altering the production of outer membrane proteins/porins), and active transport of antimicrobial agents from the bacterial cells.

In many instances, bacterial MDR results from several mechanisms; however, active efflux of the antimicrobial agent can achieve MDR. Antimicrobial efflux pumps can be categorized into many families according to the variations in their structural characteristics and energy requirements [51–54]. They enclose the following: RND (resistance-nodulation division), ABC (ATP-binding cassette), MATE (multidrug and toxic compound extrusion), MFS (major facilitator superfamily), SMR (small multidrug resistance), PACE (proteobacterial antimicrobial compound efflux), and CDF (cation diffusion facilitator) transporters [9, 53]. As far as secondary transporters go, MF is the biggest and most varied superfamily currently identified [55]. In this study, we identified variants of RND transporters, the ABC efflux complexes, the SMR family, the MF family, and the MATE superfamily [56, 57].

The RND efflux pump family consists of tripartite efflux pumps, which are composed of three main components. These components include an outer membrane protein or outer membrane factor (OMP, an inner membrane transporter protein (efflux protein), and a periplasmic adapter protein (PAP), also known as the membrane fusion protein (MFP), which acts as a connector between the OMP and the RND protein [58, 59]. In our study different mutations were detected in the RND family proteins member such as AcrAB-TolC/drug antiporter AcrB, AcrAD-TolC/drug antiporter AcrD, AcrEF-TolC/drug antiporter AcrF were identified in KP_R.Ag isolate. These proteins play a crucial role in the efflux of antimicrobial drugs, decreasing the intracellular concentration of it in the bacterial cell and consequently playing a role in AgNPs resistance acquisition. The same results were obtained by Jianhua et al. and Yang et al. after exposure of NPs on *Pseudomonas aeruginosa* [60, 61].

The Cus system is the silver resistance mechanism that can transport silver ions into the extracellular space [47, 62]. Mutations in Copper/silver efflux RND transporters proteins CusA, CusB, CusC, and CusS have been identified in KP_R.Ag isolate and have been known to be associated with silver resistance (Table 3). Mutation in CusS protein, involved in silver resistance, raises cusCFBA expression which is an efflux transporter required for silver resistance [63], and ultimately raises Ag + efflux [64].

According to genetic studies, ABC transporters are one of the most recognized protein families in prokaryotes and may be crucial to the physiology of both gram-positive and gram-negative bacteria [65]. Numerous cellular activities are mediated by bacterial ABC transporters such as MDR, biofilm formation, adhesion, attainment of necessary nutrients, formation of spores, conjugation, and toxin release [66]. Despite the fact that many vital ABC importers remain unknown, such proteins play an important role in transportation of metals and amino acids [67]. The data obtained showed that various mutations in ABC transporters were identified in both AgNPs resistant isolates, which may play a role in AgNPs resistance. Fe + 3 siderophore transport system, iron compound transporter, and ZnuA zinc transporter were identified as metal ABC transporters in KP_R.Ag isolate. Also, mutations in amino acid and protein ABC transporters were MlaB, OppF, CcmB, MdxE, LivF, SsuA, GltI, YehX, MdlB, MdxF, ModA, UrtC, methionine, phosphate/phosphonate transporter, substrate-binding protein, N-acetyl-D-glucosamine along with clusters related to maltose/polyamine/glutamine/opines/nickel and peptides. On the other hand, mutations in iron transporter and manganese transporter SitA were identified in St.L_R.Ag isolate as metal ABC transporters. PotA, PotC, PotD, MsmX, MdxE, PstB, OpuA, MdlB, OppF, OppD, YbiT, heterodimeric, methionine, and ABC excinuclease along with clusters related to maltose and basic amino acid, were marked as mutations in protein and amino acid ABC transporters in St.L_R.Ag isolate. Our results are in agreement with Jiya Jose et al. which demonstrated complete resistance of *Staphylococcus aureus* to AgNPs and induce complete mortality of *Staphylococcus aureus* when treated with Verapamil, (an efflux pump inhibitor) [68].

Different mutations were also identified as part of the efflux system in KP_R.Ag, such as MdtL, MdtM, EmrD, uncharacterized MFS transporters, and 3-(3-hydroxyphenyl) propionate transporters as part of the major facilitator superfamily (MFS) in KP_R.Ag isolate. Alterations of bacterial efflux pumps of the MFS restored the clinical utility of antimicrobial agents according to Kumar et al. and thus may play a role in AgNPs resistance in this study [69]. Mutations were also identified in Multidrug resistance proteins such as MdtG, MdtN, MdtO, MdtP, and MdtQ in KP_R.Ag isolate which had been identified in *Klebsiella pneumonia* resistance to antibiotics [70] and thus may play a role in AgNPs resistance in this study.

Permease proteins are membrane transport proteins that allow the diffusion of a specific molecule in or out of the cell. Mutations in permease proteins such as cytosine/ purine/uracil/thiamine/allantoin permease family proteins, fucose permease, and YbiR were detected in KP_R.Ag isolate which might be involved in AgNPs resistance. Contrarily, mutations in macrolide permease protein, YdaO, and AapA were detected in St.L_R.Ag isolate. OmpN, OprD, PhoE, OmpC, YaeT, OprB, FimD, outer membrane protein x precursor, and outer membrane protein/protective antigen were also recognized as outer membrane proteins in KP_R.Ag isolate alters the bacterial membrane permeability and thus plays a role in AgNPs resistance in agreement with TiO_2_ NPs resistance by Christophe Pagnout [71].

Among the Gram-negative bacteria, the most studied MATE superfamily transporters pump is the NorM efflux pump in *Neisseria gonorrhoeae* and *Vibrio cholera* [72]. The NorM efflux pump exports substrates including antimicrobial cationic compounds (quaternary ammonium compounds) and antimicrobials such as ciprofloxacin and solithromycin in *N. gonorrhoeae* [73]. In this study, mutations identified in MdtK/NorM as part of the MATE family which might be involved in AgNPs bacterial resistance [74].

According to Baharoglu et al. 2013, sub-MICs of antimicrobial reagents can elevate the horizontal transfer of antimicrobial resistance genes by increasing reactive oxygen species (ROS) formations, subsequent induction of multidrug efflux systems, and ROS-induced DNA mutagenesis [75]. In this study, long-term exposure to a sub-MIC of AgNPs altered the activity of certain proteins involved in oxidative stress and SOS response pathways. This is a global response to DNA damage in which DNA repair is induced, leading to a rise in the rate of the genome-wide mutation [76]. Mutations in variants of stress response proteins such as superoxide dismutase, universal stress protein G, DegS, DegQ, YdcZ, YdaA, YciE, HSP, GrpE, Zrap, RecF, CopD, CueR, and RcsA coregulator with RcsB were identified in KP_R.Ag isolate. Adenylate kinase, arsenate reductase, superoxide dismutase, thioredoxin reductase, MBL-fold metallohydrolase superfamily, CSP, HSP, GroEL, DnaK, DnaJ, ClpB, ClpP, ClpC, ClpX, ClpE, PhoH, YicC, FtsH, GlcU, AhpC, AhpF, and HslO were identified in St.L_R.Ag isolate [77]. Furthermore, SOS response proteins KatE and RecF in KP_R.Ag, RecA, and LexA in St.L_R.Ag had been detected in both isolates (Supplementary Tables S2 & S3), respectively. Bacteria exposed to AgNPs activate proteins responsible for safeguarding against oxidative stressors such as KatE which converts hydrogen peroxide into oxygen later on [78]. Also, it activates proteins such as superoxide dismutase which breaks down superoxide into hydrogen peroxide. Our findings are in agreement with the induced formation of free radicals produced by metals which might cause oxidative stress and trigger the SOS response [79].

According to Kamat and Kumari 2023, intracellular stress brought on by NPs modifies multidrug-resistant proteins and/or genes. This, in turn, sets off a chain reaction that results in the overexpression of membrane porin/protein genes and multidrug resistance efflux genes, ultimately fostering adaptive pathogenic evolution [3].

## Conclusion

Nowadays, NPs are applied in many fields, including agriculture, medicine, and the environment. The current work has exhibited that bacteria can quickly become resistant to the antimicrobial effects of AgNPs when they are exposed to sub-inhibitory doses of the particles regularly. Understanding the mechanism of acquiring nanoparticle resistance is crucial to overcome the current issue of resistance to NPs produced by bacteria. Resistance could be related to a mix of mutations that leads to the overexpression of several transporters, multidrug efflux pumps, and alterations of expression of enzymes and proteins incorporated in outer membrane permeability. Despite being a relatively new phenomenon, stringent regulations and rigorous adherence to the guidelines for the usage and disposal of nano wastes are needed to stop this issue from spreading as far as antibiotic resistance.

## Electronic supplementary material

Below is the link to the electronic supplementary material.

Supplementary Material 1

## Data Availability

Data of whole genome sequencing are available on NCBI under the Bioproject accession number: PRJNA1108055 and PRJNA1109764. All other data are available on request.

## Abbreviations

AgNPs: Silver nanoparticles
MIC: Minimum inhibitory concentrations
WGS: Whole genome sequencing
SNP: Single nucleotide polymorphism
BV-BRC: The Bacterial and Viral Bioinformatics Resource Center
AMR: Antimicrobial resistance
NGS: Next generation sequencing
HIV: Human immune deficiency virus
WHO: The World Health Organization
PVP: Polyvinyl pyrrolidone
FT-IR: Fourier Transform Infrared Spectroscopy
DLS: Dynamic light scattering
TEM: Transmission electron microscope
SEM: Scanning electron microscope
EDX: Energy-dispersive X-ray
MALDI-TOF MS: Matrix-Assisted Laser Desorption/Ionization Time-Of-Flight Mass Spectrometry
MHA: Muller Hinton agar
NCBI: National Center for Biotechnology Information
SPSS: Statistical Package for the Social Sciences
CLSI: The Clinical and Laboratory Standards Institute
EC.1: Escherichia coli
EC.2: Escherichia coli
KP.1: Klebsiella pneumonia
KP.2: Klebsiella pneumonia
AB: Acinetobacter baumannii
St.L.1: Staphylococcus lentus
St.L.2: Staphylococcus lentus
Ps: Pseudomonas aeruginosa
St.M: Stenotrophomonas maltophilia
St.A: Staphylococcus aureus
KP.Ag.R: AgNPs resistant Klebsiella pneumonia
St.Ag.R: AgNPs resistant Staphylococcus lentus
ABC: Adenosine triphosphate binding cassette
RND: Resistance-nodulation division
MATE: Multidrug and toxic compound extrusion
SMR: Small multidrug resistance
MFS: Major facilitator superfamily
PACE: Proteobacterial antimicrobial compound efflux
CDF: Cation diffusion facilitator
