# Supplementary material for: Genetic determinants of silver nanoparticle resistance and the impact of gamma irradiation on nanoparticle stability
Source: BMC Microbiol. 2025 Jan 13;25:18. doi: 10.1186/s12866-024-03682-x (PMC11727503; doi:10.1186/s12866-024-03682-x)
Supplement: Supplementary file 1 — Supplementary Material 1 [file 12866_2024_3682_MOESM1_ESM.docx]

**Supplementary data**

**Genetic Determinants of Silver Nanoparticle Resistance and the Impact of Gamma Irradiation on Nanoparticle Stability**

Amira M. Mahfouz¹, Walaa A. Eraqi², Hala Nour El Din El Hifnawy¹, Alaa El Din Shawky², Reham Samir², Mohamed A. Ramadan²

**¹** Department of Drug Radiation Research, Division of Biotechnology, Laboratory of Drug Microbiology, National Center for Radiation Research and Technology (NCRRT), Egyptian Atomic Energy Authority, Cairo, Egypt.

**²** Department of Microbiology and Immunology, Faculty of Pharmacy, Cairo University, Kasr El-Aini Street, Cairo11562, Egypt.

**
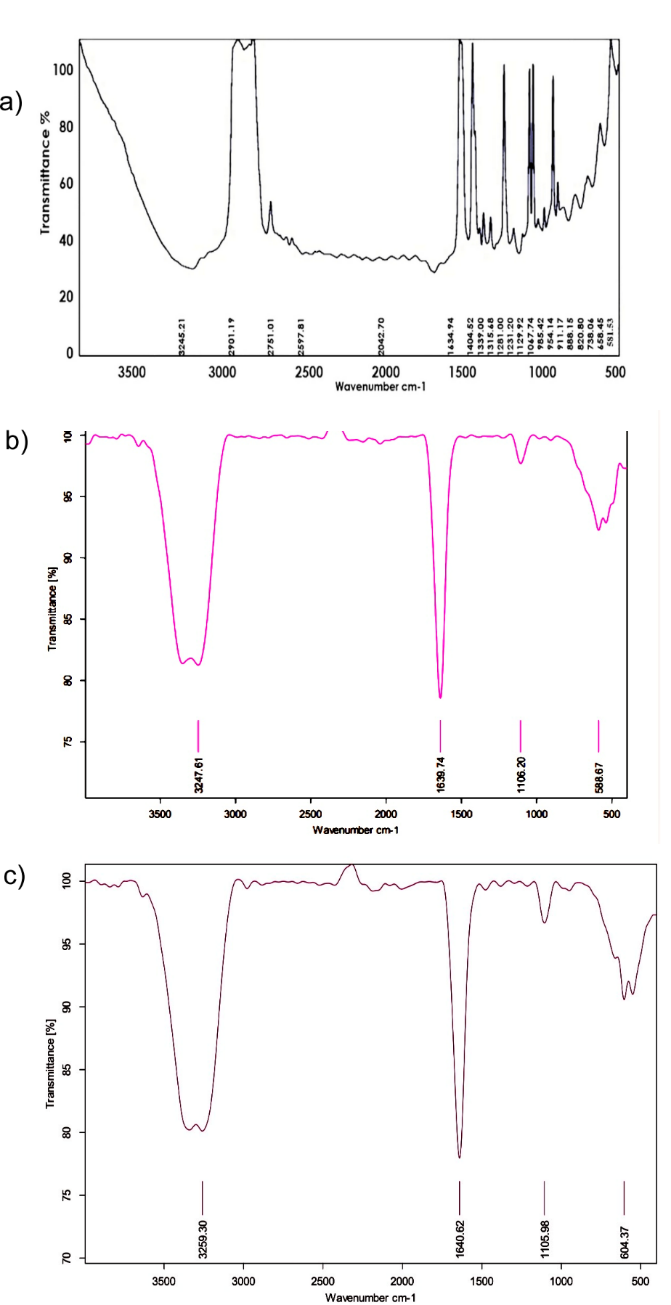
**

**Figure S1. Fourier Transform Infrared Spectroscopy (FTIR) of the prepared silver nanoparticles** a) un-irradiated silver nanoparticles showing incomplete reduction of silver ions in the solution b) Irradiated silver nanoparticles at 5kGY dose of γ irradiation showing reduced silver ions to silver nanoparticles c) Irradiated silver nanoparticles at 10 kGy dose of γ irradiation showing complete reduction of all silver ions to silver nanoparticles.

**Table S1. Identification of eleven bacterial isolates from wound infections by MALDI-TOF with score number >2.**

| ***Isolate number*** | ***Name*** |
| --- | --- |
| *1* | *Staphylococcus lentus* St.L.1 |
| *2* | *Staphylococcus lentus* St.L.2 |
| *3* | *Staphylococcus aureus* St.A |
| *4* | *Klebsiella pneumoniae* KP.1 |
| *5* | *Klebsiella pneumonia* KP.2 |
| *6* | *Pseudomonas aeruginosa* Ps.1 |
| *7* | *Pseudomonas aeruginosa* Ps.2 |
| *8* | *Escherichia coli* EC.1 |
| *9* | *Escherichia coli* EC.2 |
| *10* | *Acinetobacter baumannii* AB |
| *11* | *Stenotrophomonas maltophilia* St.M |

**Table S2. nsSNPs for KP_R.Ag isolate**

| **SNP type** | **Reference Nucleotide** | **Variant Nucleotide** | **Reference amino acid position change** | **SNP effect type** | **SNP effect impact** | **Protein /enzyme** | **Function** |
| --- | --- | --- | --- | --- | --- | --- | --- |
| Non-synonymous | Aat | AGt | Asn35Ser | Missense  Variant | Moderate | Colanic acid capsular biosynthesis activation accesory protein  **RcsA**, co-regulator with **RcsB** | Stress response |
| Non-synonymous | Cttttcgtc | TTTTTCGtc | Leu82Phe | Missense  Variant | Moderate | Inner membrane protein **YdcZ** | Stress response |
| Non-synonymous | Accgta | aACCTG | ThrVal131AsnLeu | Missense  Variant | Moderate | Universal stress protein family | Stress response |
| Non-synonymous | Gtt | Att | Val288Ile | Missense  Variant | Moderate | Outer membrane stress sensor protease  **DegQ,**  serine protease | Stress response |
| Non-synonymous | tccctc | CTCAAc | SerLeu205LeuAsn | Missense  Variant | Moderate | Outer membrane stress sensor protease  **DegS** | Stress response |
| Non-synonymous | Aaa | Gaa | Lys51Glu | Missense  Variant | Moderate | Universal stress protein G | Stress response |
| Deletion | gctgacaaaaaaaac | gcTGACAAAAAAAC | Lys68fs | Frameshift  Variant | High | Superoxide dismutase | Stress response |
| Deletion | gacaaaaaaaatctc | gaCAAAAAAATCTc | Asn227fs | Frameshift  Variant | High | Heat shock protein **HSP**  60 kDa family chaperone  **GroEL** | Stress response |
| Deletion | gcgaaaaaaaacgcg | gcGAAAAAAACGCg | Asn6fs | Frameshift  Variant | High | Heat shock protein  **GrpE** | Stress response |
| Deletion | gtgaag | GAag | Val136fs | Frameshift  Variant | High | **YciE** protein | Stress response |
| Deletion | atgaaaaaaaatgtt | atGAAAAAAATGTt | Asn84fs | Frameshift  Variant | High | **RecF** protein | Stress response |
| Non-synonymous | Tga | tgG | Ter145Trpext*? | stop lost | High | Zinc resistance-associated protein  **ZraP** | Stress response |
| Deletion | Ggcgaaaaaaat | gGCGAAAAAAT | Glu491fs | Frameshift  Variant | High | Catalase intracellular protease  **KatE** | Stress response |
| Insertion | ggcgcc | ggCGGCc | Ala364_Leu365fs | Frameshift  Variant | High | Fosmidomycin resistance protein | Stress response |
| Insertion | tatgtg | tATTGtg | Tyr34_Val35fs | Frameshift  Variant | High | Class A beta-lactamase | Stress response |
| Deletion | Gcgaaaaaaagc | GCGAAAAAAGc | Lys136fs | Frameshift  Variant | High | Universal stress protein  **YdaA** | Stress response |
| Non-synonymous | Cgc | cAc | Arg89His | Missense  Variant | Moderate | Copper resistance protein **CopD** | Stress response |
| Non-synonymous | Gcg | gAg | Ala107Glu | Missense  variant | Moderate | Copper resistance transcriptional regulator **CueR**  (MerR family) | Stress response |

**Table S3. nsSNPs of St.L_R.Ag**

| **SNP type** | **Reference Nucleotide** | **Variant Nucleotide** | **Reference amino acid** | **SNP effect Type** | **SNP effect Impact** | **Protein /enzyme** | **Function** |
| --- | --- | --- | --- | --- | --- | --- | --- |
| Deletion | aataga | aTAac | Ile638fs | Frameshift  variant | High | Tetracycline resistance, ribosomal protection type | Stress response |
| Non-synonymous | ttctataaagaaaaaaatcattttcgt | ttCTATAAAGAAAAAAATCGTTATCGA | HisPheArg174ArgTyrArg | Missense  variant | Moderate | SOS-response repressor and protease  **LexA** | Stress response |
| Non-synonymous | Aaagat | aaCGAC | LysAsp280AsnAsp | Missense  variant | Moderate | **RecA** protein | Stress response |
| Non-synonymous | Gcagac | gcAGAA | Asp12Glu | Missense  variant | Moderate | Cold shock proteins **CSP** family | Stress response |
| Non-synonymous | Tca | Gca | Ser508Ala | Missense  variant | Moderate | Heat shock HSP protein 60 kDa family chaperone **GroEL** | Stress response |
| Non-synonymous | Cacaactgc | caAAAATGT | HisAsnCys128GlnLysCys | Missense  variant | Moderate | Alkyl hydroperoxide reductase protein F  **AhpF** | Stress response |
| Non-synonymous | Acgcat | AAAAAC | ThrHis160LysAsn | Missense  variant | Moderate | Alkyl hydroperoxide reductase protein C **AhpC** | Stress response |
| Non-synonymous | gcttttttccgaaac | gcATTCTTTAAAAAT | GcATTCTTTAAAAAT | Missense  variant | Moderate | Thioredoxine reductase TR  **TrxB** | Stress response |
| Non-synonymous | cgt | cAA | Arg477Gln | Missense  Variant | Moderate | Cell division-associated, ATP-dependent zinc metalloprotease  **FtsH** | Stress response |
| Non-synonymous | Atc | GTT | Ile247Val | Missense  Variant | Moderate | proton symporter **GlcU** | Stress response |
| Non-synonymous | Gat | AAT | Asp7Asn | Missense  Variant | Moderate | Superoxide dismutase | Stress response |
| Non-synonymous | Tta | Ata | Leu7Ile | Missense  Variant | Moderate | Arsenate reductase) thioredoxin-coupled, LMWP family | Stress response |
| Non-synonymous | Gat | Aat | Asp238Asn | Missense  variant | Moderate | 33 kDa chaperonin  **HslO** | Stress response |
| Non-synonymous | Acc | GCA | Thr55Ala | Missense  Variant | Moderate | MBL-fold metallo-hydrolase superfamily | Stress response |
| Non-synonymous | Gtg | AGC | Val709Ser | Missense  variant | Moderate | Chaperone protein (ATP-dependent unfoldase)  **ClpB** | Stress response |
| Non-synonymous | Caa | Aaa | Gln145Lys | Missense  variant | Moderate | ATP-dependent Clp protease proteolytic subunit  **ClpP** | Stress response |
| Non-synonymous | Gaa | gaT | Glu609Asp | Missense  variant | Moderate | ATP-dependent Clp protease,  ATP-binding subunit  **ClpC** | Stress response |
| Non-synonymous | Gct | ATt | Ala354Ile | Missense  variant | Moderate | ATP-dependent Clp protease  ATP-binding subunit  **ClpX** | Stress response |
| Non-synonymous | Ttt | Att | Phe505Ile | Missense  variant | Moderate | ATP-dependent Clp protease,  ATP-binding subunit  **ClpE** | Stress response |
| Non-synonymous | Gaa | gaT | Glu44Asp | Missense  Variant | Moderate | Chaperone protein **DnaJ** | Stress response |
| Non-synonymous | Att | aCt | Ile47Thr | Missense  variant | Moderate | Chaperone protein **DnaK** | Stress response |
| Non-synonymous | Gtc | ATA | Val136Ile | Missense  variant | Moderate | Phosphate starvation-inducible protein **PhoH**, predicted ATPase | Stress response |
| Non-synonymous | Gac | gaA | Asp177Glu | Missense  variant | Moderate | protein **YicC** | Stress response |
| Non-synonymous | Aaa | aaT | Lys165Asn | Missense  variant | Moderate | Adenylate kinase **Ak** | Stress response |
